# Regulation Roles of Juvenile Hormone Epoxide Hydrolase Gene 2 in the Female River Prawn *Macrobrachium nipponense* Reproductive Process

**DOI:** 10.3390/cimb46120803

**Published:** 2024-11-25

**Authors:** Jisheng Wang, Mengying Zhang, Hongtuo Fu, Wenyi Zhang, Yiwei Xiong, Shubo Jin, Hui Qiao, Sufei Jiang

**Affiliations:** 1 Wuxi Fisheries College, Nanjing Agricultural University, Wuxi 214081, China; wangjs0808@163.com (J.W.); zmy20000310@163.com (M.Z.); fuht@ffrc.cn (H.F.); 2 Key Laboratory of Freshwater Fisheries and Germplasm Resources Utilization, Ministry of Agriculture and Rural Affairs, Freshwater Fisheries Research Center, Chinese Academy of Fishery Sciences, Wuxi 214081, China; zhangwy@ffrc.cn (W.Z.); xiongyw@ffrc.cn (Y.X.); jinsb@ffrc.cn (S.J.)

**Keywords:** ovarian development, molting, endocrine hormone, enzyme JHEH

## Abstract

In this study, we investigated the regulatory roles of the *juvenile hormone epoxide hydrolase* (*JHEH*) gene in the reproductive process of female *Macrobrachium nipponense*. Its total cDNA length was 1848 bp, encoding for 460 amino acids. It contained conserved domains typical of epoxide hydrolases, such as the Abhydrolase family domain, the EHN epoxide hydrolase superfamily domain, and the “WWG” and “HGWP” motifs. The qPCR results showed that the expression of *Mn-JHEH* was the highest in hepatopancreas. *Mn-JHEH* was expressed at all stages of the embryonic and larval stages. The expression of *Mn-JHEH* at different developmental periods of the ovary was positively correlated with ovarian maturation. In situ hybridization showed that it was mainly located in the cytoplasmic membrane and nucleus of oocytes. The RNA interference technique was used to study the role of *Mn-JHEH* in the process of ovarian maturation. The knockdown of *Mn-JHEH* with dsRNA in the experimental group resulted in a significant decrease in the percentage of ovaries exceeding stage O-III and the gonadal index compared with the control group. On day 14 (the second molt), the molt frequency was significantly higher in the control group than in the experimental group. The results showed that *Mn-JHEH* played an important role in ovarian maturation and molting.

## 1. Introduction

The oriental river prawn, *Macrobrachium nipponense* (class: Malacostraca, order: Decapoda), is an important commercial aquaculture species in China, with a production of about 272,592 tons in 2016 and an annual output value of more than 20 billion RMB [1,2,3]. This species has been extensively cultured in China due to its short breeding cycle, strong adaptability, low susceptibility to disease, and high nutritional value [4,5]. However, when the female prawn enters the breeding period, the gonadal maturation cycle is greatly shortened and the reproduction rate is rapid, resulting in multi-generation reunion, high reproductive density, and the general miniaturization of the prawn, which seriously affects the commercial production value and economic benefits of prawn culture [6,7]. Therefore, it is of great significance to investigate the reproductive regulation mechanism of *M. nipponense*. Many genes were found with sexual rapid maturation [7]. An analysis of the hepatopancreas transcriptome in female *M. nipponense* adult ovaries during stages O-I to O-V identified that the “insect hormone synthesis” signaling pathway was closely linked to ovarian development based on KEGG enrichment results [5]. Notably, juvenile hormone epoxide hydrolase exhibited significant expression in this pathway. Therefore, the role of *JHEH* in reproductive regulation was further investigated. 

The juvenile hormone (JH) is an endocrine hormone that plays an important role in the growth, development, metamorphosis, and reproduction of crustacea and insects [8,9,10,11]. The metabolism of the JH is regulated by various genes including juvenile hormone esterase (JHE), juvenile hormone methyltransferase (JHAMT), and juvenile hormone epoxide hydrolase [12,13]. JHEH is a key enzyme in the JH metabolism, catalyzing the opening of the epoxide ring in the JH to form juvenile hormone diol (JHD). Additionally, it can degrade juvenile hormone acid (JHA) to juvenile hormone acid diol (JHAD) [14]. Touhara et al. [15] purified JHEH protein in *Manduca sexta* eggs [15], and then the cDNA of *JHEH* was obtained from various insect species, such as *Ctenocephalides felis* [16], *Bombyx mori* [17], *Tribolium castaneum* [18], and *Heliothis virescens* [19]. Many studies have demonstrated that *JHEH* is involved in the growth, development, and molting of insects. Tusun et al. found that after silencing the juvenile hormone epoxide hydrolase gene by RNA interference technology, the survival rate of *Apolygus lucorum* nymphs was significantly reduced, together with a molting block [14]. In *Nilaparvata lugens*, it has been observed that RNA interference targeting *JHEH* can impact wing-type differentiation [20]. In crustaceans, the immune-related function of *JHEH* in *Penaeus vannamei* has been investigated by analyzing the transcriptional levels of *JHEH* after attack by Gram-positive and Gram-negative bacteria. The results indicate that *JHEH* plays a crucial role in protecting *Penaeus vannamei* against bacterial infections [12]. However, the function of *JHEH* in crustaceans in relation to reproduction regulation remains unclear. 

In this study, the sequence characteristics and phylogenetic relationships of *JHEH* in *M. nipponense* were analyzed using bioinformatics methods. The expression of the *JHEH* gene was investigated by qPCR in different tissues at different stages of the ovary and embryo development of *M. nipponense*. RNAi technology was used to silence the *JHEH* gene to explore its expression in the ovaries after silencing and its effect on molting and ovarian maturation in *M. nipponense*. It has been demonstrated that *JHEH* plays an important role in regulating the reproduction of *M. nipponense*.

## 2. Materials and Methods

### 2.1. Experimental Prawns

Healthy female *M. nipponense* were obtained from the Freshwater Fisheries Research Center of the Chinese Academy of Fisheries Sciences, Wuxi, Jiangsu Province, China. No endangered or protected species were involved in the experiment. All experimental protocols and methods were approved in September 2022 (authorization No. 20220901005) by the Animal Care and Use Ethics Committee in the Freshwater Fisheries Research Center (Wuxi, China). Female prawns were cultured in an indoor recirculating aquaculture system (recirculating glass aquarium tanks with a volume of about 100 L), and the water temperature was maintained at 27 ± 1°C. The pH was 6.7 to 7.1, the dissolved oxygen content was 5.5 to 6.9 mg/L, and the ammonia nitrogen content was 0.25 to 0.28 mg/L. The prawns were acclimated to laboratory conditions for at least a week before the experiment, during which time they were fed twice daily with paludina. We cleaned up the leftovers and changed the water during breeding.

### 2.2. Sample Collection

Thirty healthy and viable adult female *M. nipponense* weighing 0.56 ± 0.13 g were used for tissue expression studies. Tissues collected for analysis included the eyestalk (E), brain (BR), heart (H), hepatopancreas (He), gills (G), ovary (O), and muscle (M), with six samples per tissue (n = 6). Fifty adult female *M. nipponense* prawns (BW ± SD: 0.78 ± 0.24 g) at different stages of ovarian development were used for ovarian expression profiling. According to a previous study [6,21], the five developmental stages of the ovary were defined according to the color of the ovary. The stage categories of embryos were also defined according to a previous study. Larval (L) and post-larval (PL) developmental stages were sampled every 5 days across 9 time points: L1, L5, L10, L15, PL1, PL5, PL10, PL15, and PL25. All samples were stored in liquid nitrogen.

### 2.3. Total RNA Extraction and cDNA Synthesis

Total RNA was extracted from prawn tissues using RNAiso Plus reagents (TaKaRa, Tokyo, Japan) following the manufacturer’s instructions, and the quality was verified through 1% agarose gel electrophoresis. Subsequently, RNA concentration was determined using a Nanodrop one spectrophotometer (Thermo Fisher Scientific, Waltham, MA, USA). For cDNA synthesis, total RNA was used as a template, and the M-MLV reverse transcriptase (TaKaRa, Tokyo, Japan) kit was used according to the manufacturer’s instructions. All cDNA samples were stored at −20 °C until further use.

### 2.4. Gene Cloning and Sequence Analysis of Mn-JHEH

Partial sequences of *JHEH* were obtained from the hepatopancreas transcriptome of female *M. nipponense* adult ovaries from development stages O-I to O-V of development [5]. The partial sequences of *JHEH* were cloned and validated by PCR. The cDNA 3′ and 5′ ends sequence of *JHEH* was cloned using the 3′RACE kit and 5′RACE kit (TaKaRa, Shiga, Japan), according to the manufacturer’s instructions. The full-length *JHEH* sequence was obtained by combining partial sequences of the hepatopancreas transcriptome library of *M. nipponense* with the 3′ and 5′race products using DNAMAN 6.0 software (Lynnon Biosoft, San Ramon, CA, USA). The identified sequence was subsequently confirmed using PCR and then sequenced. Primers used were designed by the prime designing tool (https://www.ncbi.nlm.nih.gov/tools/primer-blast/) (accessed on 16 April 2022). All primer sequences are listed in Table 1.

Molecular weight (MW) and the theoretical isoelectric point (pI) of *Mn-JHEH* were calculated using ProtParam on the ExPASy website (http://web.expasy.org/protparam/) (accessed on 16 April 2022). The open reading frame (ORF) of *Mn-JHEH* was predicted using the ORF Finder program provided by the National Center for Biotechnology (NCBI) (http://ncbi.nlm.nih.gov/gorf/gorf.html) (accessed on 16 April 2022). Protein domain prediction was performed using Conserved Domain Searches at NCBI (https://www.ncbi.nlm.nih.gov/Structure/cdd/wrpsb.cgi) (accessed on 16 April 2022). Multiple alignments of amino acid sequences of *Mn-JHEH* with homologs from other species were performed using DNAMAN 6.0 software. The phylogenetic tree was constructed by the neighbor-joining (NJ) method in MEGA 7.0 software (Mega Limited, Auckland, New Zealand).

### 2.5. In Situ Hybridization

The subcellular localization of *Mn-JHEH* at stage IV of ovarian development was examined by ISH. The ovary and hepatopancreas from *M. nipponense* at stage IV of ovarian development were dissected out and soaked in 4% paraformaldehyde (PBS, pH 7.4) for 12 h at 4 °C. A Zytofast PLUS CISH implementation kit (code No. T-1061-40, ZytoVision GmBH, Bremerhaven, Germany) was used for tissue fixation and paraffin embedding. The specific experimental steps for in situ hybridization were performed by referring to the instructions for the CISH technique. The antisense and sense probes used in achromogenic ISH were designed by Primer5 software (Premier Technologies Inc., San Francisco, CA, USA) based on the cDNA sequence of *Mn-JHEH*. Both sense and antisense probes with digoxin signals were synthesized by Sangon Biotech. More details have been reported in previous studies [4]. The sense probe was prepared for the experimental group, and the probe sequence was 5’-ATGGTATCCTGTCCATGAGGAAGGTTTCAGAC, whereas the antisense strand probe (5’-GTCTGAAACCTTCCTCATGGACAGGATACCAT) was prepared for the control group. HE was the blank control group, which was stained with hematoxylin–eosin. The sense probe was used for the experimental group. The antisense probe was used for the negative control group. All slides were examined using a light microscope.

### 2.6. RNA Interference (RNAi)

RNAi technology was used to study the function of *Mn-JHEH* in the regulation of ovarian maturation. The green fluorescent protein (*GFP*) gene was used as the control gene. The specific primers (Table 1) used to synthesize the dsRNA were designed with the online Snap Dragon software (https://www.flyrnai.org/cgi-bin/RNAi_find_primers.pl) (accessed on 22 July 2022) and synthesized using the Transcript Aid™ T7 High Yield Transcription kit (Fermentas Inc., Rockville, MD, USA) following the manufacturer’s protocol. The concentration of synthesized dsRNA was determined using the Nanodrop one spectrophotometer. The dsRNA was diluted to 4 µg/g with DEPC water, and then the quality of dsRNA was assessed by means of agarose gel electrophoresis, followed by storage in an ultra-low-temperature refrigerator at −80°C.

At the beginning of the study, most female ovaries were in stage IV and were used for RNAi experiments. Three hundred healthy female (n = 300) prawns (0.58 ± 0.16 g) in ovary stage IV were selected and randomly assigned to either the experimental or control groups, with 3 replicates of 50 individuals (n = 50) in each group. The water temperature was maintained at 27 ± 1 °C, and the prawns were fed once in the morning and once in the evening. Prawns in the control and experimental groups were injected with ds-GFP and ds-JHEH at a dose of 4 µg/g body weight (b.w.), respectively. At 1, 4, and 7 days after injection, 9 prawns (3 biological replicates) were randomly collected from each group, and their ovaries were dissected and immediately frozen in liquid nitrogen for the analysis of interference efficiency. On days 1, 7, 14, and 20 after injection, 9 prawns (3 biological replicates) were randomly collected from each group. Ovaries were dissected, weights and gonadal weights were recorded, and the GSI was determined as previously described [7] (GSI = gonadal weight/body weight × 100%). Ovarian samples from days 1 and 7 were shared, and GSI was measured and then analyzed for interference efficiency. The ovary samples were then immediately frozen in liquid nitrogen and stored at −80 °C. Ovarian development was observed daily, focusing on female prawns past stage O-III (yolk accumulation stage), and the ovarian development period of each individual was recorded. The molting frequency of each parallel line was also recorded daily to calculate the cumulative number of molts. Molting frequency was determined by the following formula: molting frequency = (Nm/Ns)/D, where Nm represents the total number of molts, Ns denotes the number of prawns in the aquarium, and D stands for the number of experimental days [22]. At the end of the experiment, one prawn at stage O-IV was randomly selected from each of the experimental and control groups; their ovaries were dissected and prepared as paraffin sections for comparison.

### 2.7. qPCR and Statistical Analysis

The *EIF* gene (eukaryotic translation initiation factor 5A) was used as a reference gene (Table 1). Previous studies had specific methodological steps [23]. The relative expression of the genes was calculated using the 2^−∆∆Ct^ method [24]. All quantitative data conformed to the homogeneity of variance and the normal distribution, and all the data were presented as mean ± standard deviation. Statistical significance was determined using a one-way analysis of variance (ANOVA) and Duncan’s multiple range tests in SPSS 24.0 software. An independent sample *t*-test was used to compare the experimental and control groups, with *p* < 0.05 considered statistically significant.

## 3. Results

### 3.1. Sequence Analysis of Mn-JHEH

**Figure 1:**
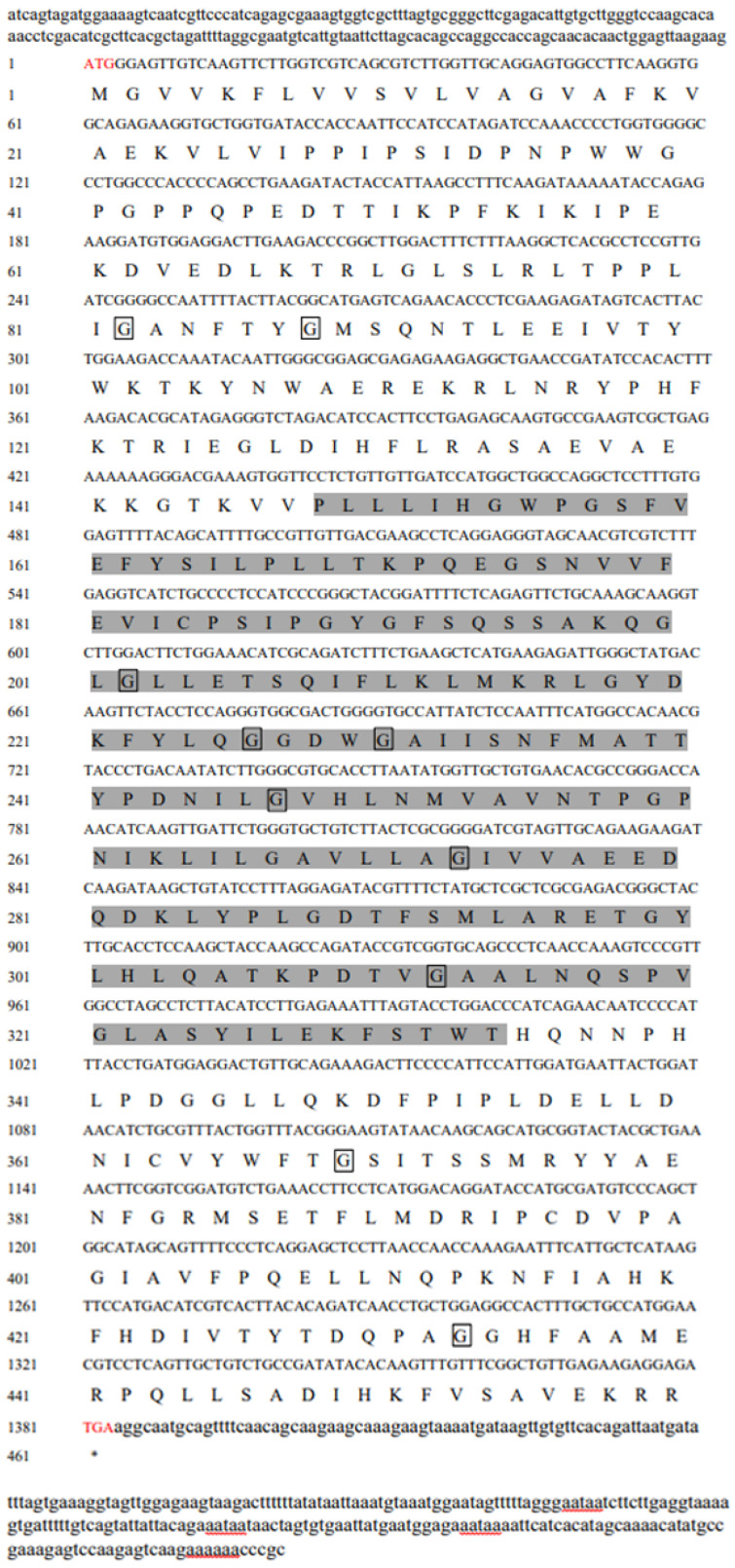
Full-length cDNA and deduced amino acid sequence of *Mn-JHEH*. Highlighted in red are the start codon ATG and the stop codon TGA. The typical Abhydrolase family sequence is highlighted in the gray box. The N-myristoylation sites are boxed. The asterisk (*) indicates the stop codon TAG in the amino acid sequence. Red underlining indicates the tail signal and poly(A).

**Figure 2:**
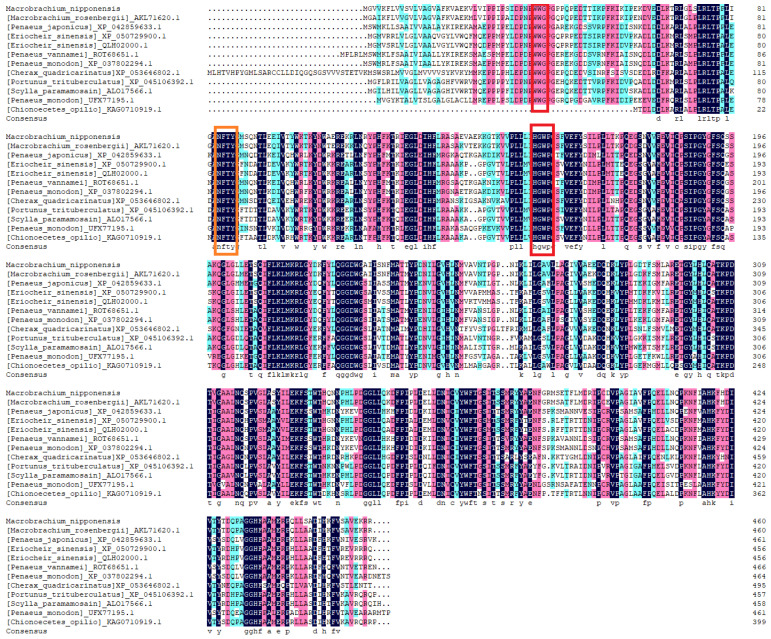
Multiple sequence alignment of *Mn-JHEH* and *JHEH* from other species. From top to bottom, the species are *Macrobrachium nipponense*, *Macrobrachium rosenbergii*, *Penaeus japonicus*, *Eriocheir sinensis*, *Penaeus vannamei*, *Penaeus mondon*, *Cherax quadricarinatus*, *Portunus trituberculatus*, *Scylla paramamosain*, *Penaeus mondon* and *Chionoecetes opilio*. The conserved “WWG” and “HGWP” motifs are marked by red boxes. N-glycosylation sites are marked by orange boxes. The black area indicates that the amino acid consistency was equal to 100%, the pink area indicates that the amino acid consistency was ≥75%, and the green area indicates that the amino acid consistency was ≥50%.

Based on the hepatopancreas transcriptome of female *M. nipponense* adult ovaries from the O-I to O-V stages of development, the *JHEH* gene was cloned using DNA as a template and designated as *Mn-JHEH* (GenBank: OR264358). The *Mn-JHEH* gene has a full-length cDNA of 1848 bp with an ORF of 1383 bp that encodes a protein of 460 amino acids, The 5′ non-coding region (5′UTR) contained 184 bp and the 3′UTR contained 281 bp. The predicted molecular formula was C2362H3660N598O653S13. The MW was 51.29 kDa, the theoretical pI was 6.61, and the total number of negatively charged amino acids (Asp+Glu) and positively charged amino acids (Arg+Lys+His) were 46 and 55, respectively. The deduced *Mn-JHEH* protein contained a typical Abhydrolase family domain in the 148–334 residues and the EHN epoxide hydrolase superfamily domain in residues 51–162 of the protein. The deduced *Mn-JHEH* protein has structural features unique to the epoxide hydrolase family, namely, a sequence catalytic triplet and Asp228, His 249, and Asp423 residues. The deduced *Mn-JHEH* protein was predicted to contain ten potential N-myristoylation sites (amino acids 82, 88, 207, 226, 230, 247, 273, 312, 369, and 433), as shown in Figure 1. Sequence analysis of the *Mn-JHEH* protein revealed that it contained conserved “WWG” and “HGWP” motifs (Figure 2).

### 3.2. Similarity Comparison and Phylogenetic Analysis

Multiple sequence alignment showed a similarity of Mn-JHEH with JHEH from Macrobrachium rosenbergii, Penaeus japonicus, Eriocheir sinensis, Penaeus vannamei, Penaeus mondon, Portunus trituberculatus, Cherax quadricarinatus, and Scylla paramamosain of 94.57%, 67.10%, 66.96%, 65.65%, 65.14%, 67.39%, 66.51%, and 66.74%, respectively (Figure 2). The similarities between Mn-JHEH and the above crustaceans were all greater than 65%, indicating that JHEH is highly conserved in crustaceans.

**Figure 3:**
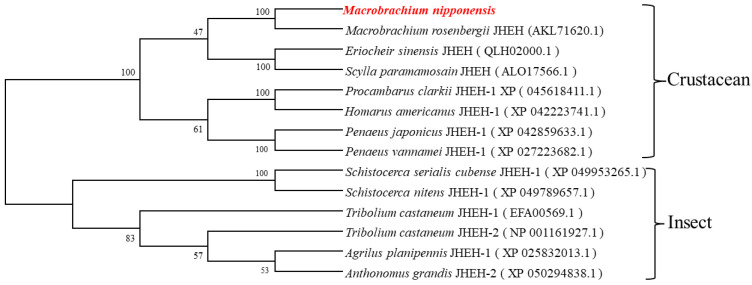
Neighbor-joining phylogenetic tree analysis of *Mn-JHEH* and other known *JHEH* proteins. The red font is the experimental prawn in this study—*Macrobrachium nipponense*. The sequence numbers in brackets indicate the GenBank accession numbers. The numbers on the branches indicate the bootstrap values (%).

Using MEGA 7.0 software (Mega Limited, Auckland, New Zealand), the amino acid sequence of the *Mn-JHEH* gene and the deduced amino acid sequence of the *Mn-JHEH* gene of other species were used for phylogenetic analysis. Phylogenetic tree analysis revealed two major clades, crustaceans and insects. Phylogenetic tree analysis showed that the *JHEH* of *M. nipponensis* clustered with the *JHEH* of *Macrobrachium rosenbergii* in the crustacean clade (Figure 3).

### 3.3. Tissue-Specific Gene Expression of Mn-JHEH

**Figure 4:**
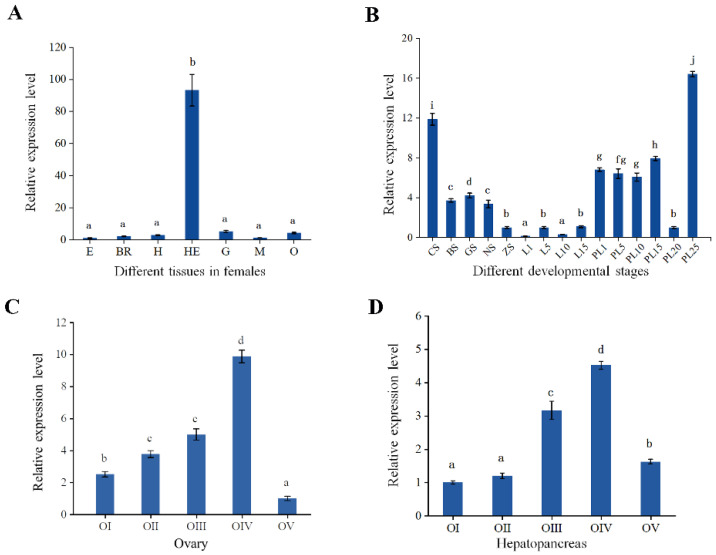
Expression analysis of *Mn-JHEH*. (**A**) Expression pattern in different tissues; (**B**) expression pattern in developmental stages; (**C**) expression patterns in the ovary at different ovarian stages; (**D**) expression pattern in the hepatopancreas at different ovarian stages. (**A**) E, eyestalk; BR, brain; H, heart; He, hepatopancreas; G, gill; M, muscle; O, ovary. (**B**) CS, cleavage stage; BS, blastocyst stage; GS, gastrulation stage; NS, nauplius stage; ZS, zoea stage; L1, the 1st-day larvae after hatching; L5, the 5th-day larvae after hatching; L10, the 10th-day larvae after hatching; L15, the 15th-day larvae after hatching; PL1, post-larval stage of the first day; PL5, post-larval stage of five days; PL10, post-larval stage of ten days; PL15, post-larval stage of fifteen days; PL-25, post-larval stage of twenty-five days. (**C**,**D**) O-I, undeveloped stage; O-II, developing stage; O-III, nearly ripe stage; O-IV, ripe stage; and O-V, spent stage. Data are presented as the mean ± SD (n = 6). Different lowercase letters indicate significant differences (*p* < 0.05). There was no difference between the same lowercase letters and significant differences between different lowercase letters.

The expression results of *Mn-JHEH* in different tissues of female *M. nipponense* are shown in Figure 4A. *Mn-JHEH* was expressed in all tissues, with the strongest expression in the hepatopancreas (*p* < 0.05), followed by the gill and ovary, and there was a weak expression in the eye stalk, brain, heart, and muscle.

The expression results of *Mn-JHEH* in embryonic and larval stages are shown in Figure 4B. The expression of *Mn-JHEH* decreased during embryonic developmental stages (from the CS–ZS stages) (*p* < 0.05). Low expression levels were maintained from the 1st day (L1) to the 15th day (L15) after hatching. The expression of *Mn-JHEH* increased from the 1st day after metamorphosis (PL1), was significantly down-regulated on the 20th day after metamorphosis (*p* < 0.05) and was significantly up-regulated on the 25th day after metamorphosis (*p* < 0.05).

The expression patterns of *Mn-JHEH* in the ovary (Figure 4C) and hepatopancreas (Figure 4D) were further analyzed at five stages of ovarian development. The results showed that the expression of *Mn-JHEH* mRNA in the ovary and hepatopancreas tended to increase at stages I-IV, reached a maximum at stages O-IV (*p* < 0.05), and declined significantly at stages IV-V (*p* < 0.05) as the ovary developed. The expression of *Mn-JHEH* in the ovary and hepatopancreas was positively correlated with ovarian development.

### 3.4. Subcellular Localization of Mn-JHEH

**Figure 5:**
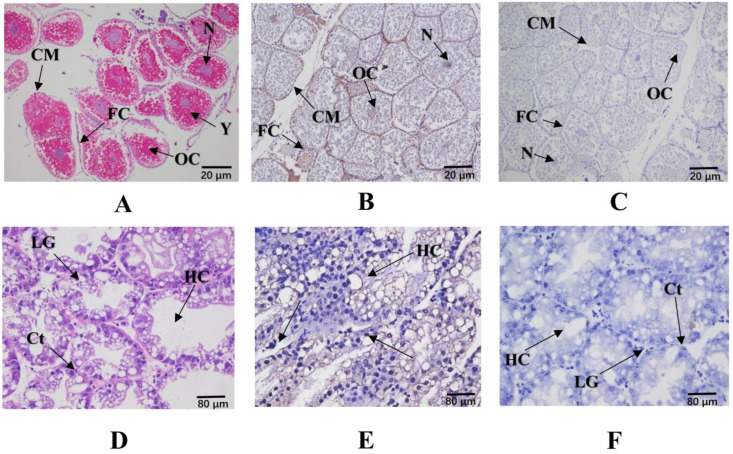
The location of the *JHEH* gene was detected in the ovary and hepatopancreas of *M. nipponense* following ISH. (**A**) Blank control for the ovary group; (**B**) positive experimental group for the ovary group; (**C**) negative control for the ovary group; (**D**) blank control for the hepatopancreas group; (**E**) positive experimental group for the hepatopancreas group; (**F**) negative control for the hepatopancreas group. OC: oocyte; N: nucleus; CM: cytoplasmic membrane; Y: yolk granule; FC: follicle cell; LGs: lipid granules; HCs: hepatocytes; Cts: connective tissues. The corresponding cells and tissues above are indicated by arrows in the figure. Scale bars: 100× (O); 400× (He).

The position of *Mn-JHEH* in the ovary and hepatopancreas was located by ISH (Figure 5). The results showed that there were obvious *Mn-JHEH* signals mainly distributed in the cell membrane and nucleus in the ovarian development stage III of *M. nipponense.* The signal was visualized in all of the oocyte types, including yolk granules, the nucleus, and the cytoplasmic membrane. The *Mn-JHEH* signal was present in the hepatopancreas of the ovarian development stage IV of *M. nipponense*, which was mainly distributed in connective tissues. 

### 3.5. Functional Analysis of Mn-JHEH in Ovarian Maturation Regulation

#### 3.5.1. Effect of RNAi on the Expression of Mn-JHEH

**Figure 6:**
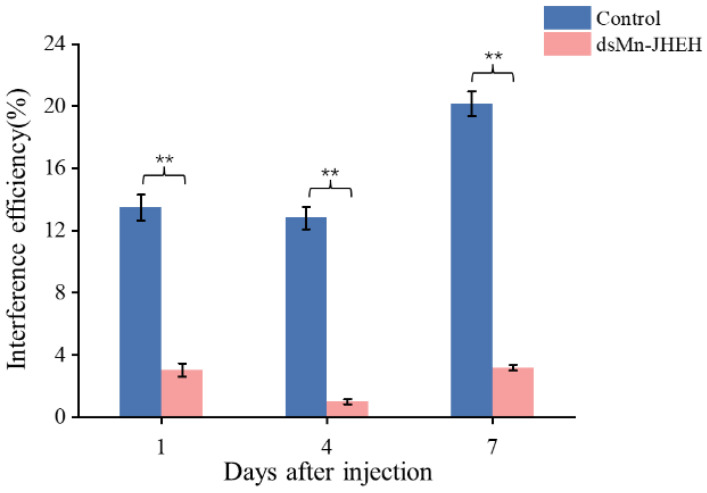
Expression levels of *Mn-JHEH* in the ovaries of the *Macrobrachium nipponense* after injection of ds-GFP and ds-JHEH. Data are shown as the mean ± SD (n = 6). ** indicates a highly significant difference (*p* < 0.01).

The function of *Mn-JHEH* regulating ovarian maturation was studied by the RNAi technique. The expression of *Mn-JHEH* in prawn ovaries after knockdown of the experimental and control groups with ds-JHEH and ds-GFP, respectively, is shown in Figure 6. Compared with the control group, the expression of *Mn-JHEH* in the experimental group was down-regulated by 80.41%, 90.75%, and 84.18% on days 1, 4, and 7 after injection, respectively (*p* < 0.01).

#### 3.5.2. Effect of RNAi on the Ovarian Maturation of Mn-JHEH

**Figure 7:**
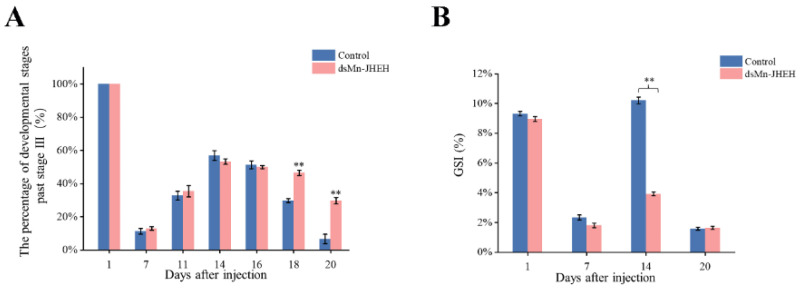
The function of *Mn-JHEH* in the ovary. (**A**) Changes of the cumulative proportion of ovary stage III of female *M. nipponense* after RNAi; (**B**) changes of the GSI (%) of female *M. nipponense* after RNAi. Data are shown as the mean ± SD (n = 6). ** indicates the significance of the differences (*p* < 0.01).

To determine the effect of *JHEH* gene knockout on ovarian maturation in *M. nipponense*, the cumulative proportion of ovaries reaching stage O-III (Figure 7A) and the gonadosomatic index (GSI) were also recorded for the two groups after injection (Figure 7B). At the beginning of the experiment, female prawns at stage IV of ovarian development were selected, and the proportion of ovarian stage O-III was 100% (*p* > 0.05). Most of the ovaries of the prawn developed to stage II after 7 days in both the experimental and control groups. On day 14, most of the prawn ovaries had developed to stage IV in the control group, while the experimental group mostly developed to stage III. The ovaries of the control group began to empty gradually after the 16th day and started to enter the next round of development, and almost all of them were emptied by the 20th day. In the experimental group, the ovaries began to empty gradually after day 18, while most of the ovaries were still in the previous round of development (*p* < 0.01). The GSI data are shown in Figure 7B. On days 1, 7, and 20 after injection, there was no significant difference in the GSI between the control group and the experimental group (*p* > 0.05). On day 14, the GSI of the control group (10.21%) was significantly different from that of the experimental group (3.92%) (*p* < 0.01).

#### 3.5.3. Effect of RNAi on the Molting Frequency of Mn-JHEH

**Figure 8:**
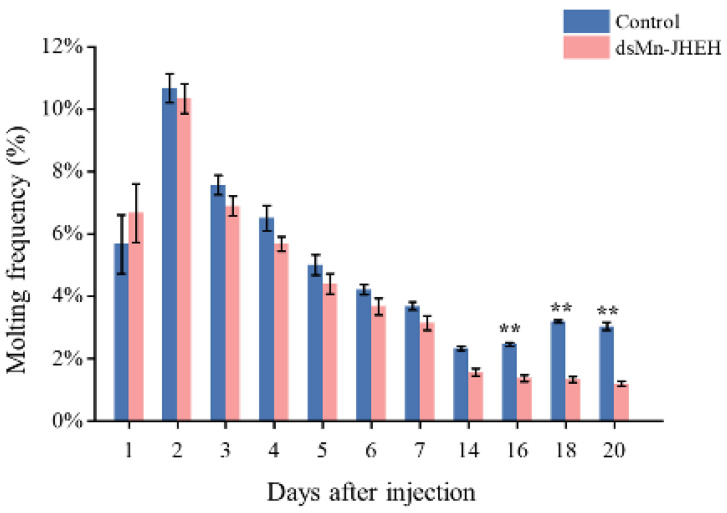
The molting frequency (%) of female *M. nipponense* after injection with *Mn-JHEH* dsRNA. Data are shown as the mean ± SD (n = 6). ** indicates the significance of the differences (*p* < 0.01).

Figure 8 shows the molting frequency of *M. nipponense* in both the experimental and control groups following a successful *Mn-JHEH* knockout. During the initial molt from the first day to the seventh, no significant difference was observed between the experimental group and the control group (*p* > 0.05). However, a second round of molting occurred in the control group on day 14, resulting in a significantly higher frequency of molting compared to the experimental group (*p* < 0.05).

## 4. Discussion

The juvenile hormone (JH) modulates a variety of development stages and reproductive maturation in insects [11,25,26]. Previous studies have suggested that *JHEH* was a multifunctional enzyme that was pivotal in the degradation of the JH titer during insect development [20,27,28]. In this study, the *JHEH* gene was identified from the hepatopancreas transcriptome of *M. nipponense* [5], and a cDNA containing a full open reading frame of 1383 bp was obtained. Protein functional domain prediction revealed that *Mn-JHEH* contains a typical Abhydrolase family domain and the EHN epoxide hydrolase superfamily domain, which is typical of the α/β-hydrolase domain protein family and also includes the two conserved motifs “WWG” and “HGWP”. These structures are typical of epoxide hydrolases and are necessary for the function of epoxide hydrolases. This was consistent with the results of *JHEH* studies on *Drosophila melanogaster* [29], *Aedes aegypti* [30], and *Lymantria dispar* [31]. The phylogenetic tree analysis showed that the amino acid sequence of CH7D was clustered with that of *Macrobrachium rosenbergii*, which was consistent with the traditional classification.

To validate the significance of *Mn-JHEH* in the reproductive regulation of *M. nipponense*, qPCR was conducted to determine the expression profile of this gene in different tissues, different stages of the ovary, and different life stages. The *Mn-JHEH* gene was observed to be expressed in all tissues, with the highest expression levels observed in the hepatopancreas. The hepatopancreas plays a crucial role in crustaceans, where it is involved in food digestion, absorption, immunity, and other physiological processes [32,33]. Studies have demonstrated that hepatopancreas and ovarian development are also closely related, and the vitellogenin (Vg) of crustaceans is mainly synthesized in the hepatopancreas [34]. In addition, high expression was also detected at the cleavage stage (CS) and day 25 after metamorphosis. The expression of *JHEH* was elevated in the early stage of development due to its involvement in biological growth and development, and it can promote the division and differentiation of early embryonic cells [31]. Consistent with the findings from *Macrobrachium rosenbergii* research, the increase in *JHEH* gene expression in late development can facilitate the degradation of the JH during the process of metamorphosis [35]. Given the high expression levels of *Mn-JHEH* in the hepatopancreas, examined its expression patterns in both the hepatopancreas and ovaries at different stages of ovarian development. The results showed a continuous increase in *Mn-JHEH* expression from stage I to stage IV, with the highest expression at stage IV, which was the gonadal maturity stage, indicating that the gene was closely related to ovarian maturation. The ISH results showed that a significant *Mn-CH7D* signal was detected in both the ovary and hepatopancreas. It is well distributed around cell membranes and intercellular spaces. These results suggest that *Mn-CH7D* played a specific role in ovarian maturation. RNA interference (RNAi) techniques were employed to investigate the contribution of *Mn-JHEH* in regulating reproduction in *M. nipponense*. The experimental group exhibited a significant reduction in the expression of the *Mn-JHEH* gene compared to the control group, with levels decreasing by 90.75% and 84.18% on days 4 and 7 after the application of RNAi, respectively. These results demonstrated that dsMnJHEH injection on day 4 effectively decreased the expression of *Mn-JHEH* in the ovaries of *M. nipponense*. After the successful silencing of *Mn-JHEH*, gonad development was significantly inhibited in the experimental group. On day 18 after injection of dsMnJHEH, the gonad development in the control group progressed to the next round, while the experimental group remained in the previous round. These results indicated that silencing *Mn-JHEH* effectively inhibited the gonadal development in *M. nipponense*. According to homology analysis, crustaceans may have similar regulatory mechanisms as insects. Correspondingly, research has demonstrated that juvenile hormones secreted by the pharyngeal body of insects typically induce the synthesis of Vg in the fat body [36,37,38]. In *Tribolium castaneum*, the JH regulates Vg synthesis through an insulin-like peptide signaling pathway [39]. Vitellogenin can promote oocyte and embryo development. Furthermore, studies have found that silencing *JHEH* in diapause insects can promote ovarian development and prepare for reproductive diapause [40]. In the RNAi experiment, the GSI was calculated to further demonstrate the relationship between *JHEH* and gonad maturation. On day 14, the GSI of the experimental group injected with *Mn-JHEH* dsRNA was significantly lower than that of the control group. The results confirmed that injection of *Mn-JHEH* dsRNA could effectively inhibit ovarian maturation, which highlighted the crucial role of *JHEH* in gonad maturation.

In addition, during the experiment, the amount of molting was recorded, and the frequency of molting was counted, revealing that molting was significantly inhibited after successful silencing of *Mn-JHEH*. Similarly, studies in *L. decemlineata* found that knockdown *JHEH* using RNAi delayed larval development and significantly damaged adult emergence [41]. Moreover, Zhang et al. treated *Litopenaeus vannamei* with juvenile hormone analogs and found that the molting of the prawn was delayed [42]. In addition, a similar finding was found in *Apolygus lucorum*, where injection with dsRNA successfully knocked out the target gene *JHEH*, resulting in a molting block phenomenon [15]. These results strongly suggested that the *Mn-JHEH* gene plays an important role in the molting and ovarian maturation of *M. nipponense* and will be useful in further studies of the reproductive regulation mechanism of crustaceans. Further studies will investigate the presence of the JH hormone in *M. nipponense* and its regulatory relationship with *JHEH*, aiming to enrich the molecular mechanism of ovarian maturation in crustaceans. 

## 5. Conclusions

In general, the *Mn-JHEH* gene was identified from the hepatopancreas transcriptome at different stages of *M. nipponense* ovarian development, where abundant transcription expression has been detected in the hepatopancreas. Interference experiments indicate that *Mn-JHEH* plays an important role in the molting and ovarian development of *M. nipponense*. This study further enriches the molecular mechanism of reproductive regulation in *M. nipponense* and provides new information regarding the reproductive regulation in *M. nipponense*. It also provided a theoretical basis for rapid sexual ripening in production. Furthermore, the research team identified numerous genes associated with accelerated sexual maturation in the early stage of development and conducted extensive investigations into their functionalities. The results provided the potential to enable molecular breeding through the utilization of gene editing technology.

## Figures and Tables

**Table 1:** Primers involved in studying gene expression in female prawn Macrobrachium nipponense in the reproductive process.

Primer’s Name	Sequence (5’-3’)	Usage
*JHEH* F1	GTTGTCAAGTTCTTGGTCGTCAG	ORF
*JHEH* R1	CATGAGCTTCAGAAAGATCTGCG	ORF
*JHEH* F2	GATCGGGGCCAATTTTACTTACG	ORF
*JHEH* R2	CATGCTGCTTGTTATACTTCCCG	ORF
*JHEH* F3	TCTGAAACCTTCCTCATGGACAG	ORF
*JHEH* R3	TCTTCTCAACAGCCGAAACAAAC	ORF
*JHEH* F3-1	ATCTTCGGGGAGAGTGCG	3’Race
*JHEH* F3-2	CGAACTCCGACCACCAGA	3’Race
3’RACE Outer	TACCGTCGTTCCACTAGTGATTT	3’Race
3’RACE Inner	CGCGGATCCTCCACTAGTGATTTCACTATAGG	3’Race
*JHEH* F5-1	CCCCGATCAACGGAGGCGTG	5’Race
*JHEH* F5-2	GGCCCCACCAGGGGTTTGGA	5’Race
5’RACE Outer	CATGGCTACATGCTGACAGCCTA	5’Race
5’RACE Inner	CGCGGATCCACAGCCTACTGATGATCAGTCGATG	5’Race
*JHEH* F	GTTGCAGAAAGACTTCCCCATTC	qPCR
*JHEH* R	CTTCTCAACAGCCGAAACAAACT	qPCR
*EIF* F	CATGGATGTACCTGTGGTGAAAC	qPCR
*EIF* R	CTGTCAGCAGAAGGTCCTCATTA	qPCR
*JHEH* dsF	TAATACGACTCACTATAGGGGAGCGAGAGA	dsRNA
*JHEH* dsR	AGAGGCTGAA	dsRNA
*GFP* dsF	TAATACGACTCACTATAGGGACGAAGACCTT GCTTCTGAAG	dsRNA
*GFP* dsR	TAATACGACTCACTATAGGGAAAGGGCAGAT TGTGTGGAC	dsRNA

## Data Availability

Data information will be provided upon request.

## References

[1] Jin S.B., Hu Y.N., Fu H.T., Sun S.M., Jiang S.F., Xiong Y.W., Qiao H., Zhang W.Y., Gong Y.S., Wu Y. (2020). Analysis of testis metabolome and transcriptome from the oriental river prawn (*Macrobrachium nipponense*) in response to different temperatures and illumination times. Comp. Biochem. Physiol. Part D Genom. Proteom..

[2] Jin S.B., Fu H.T., Jiang S.F., Xiong Y.W., Qiao H., Zhang W.Y., Gong Y.S., Wu Y. (2022). RNA Interference Analysis Reveals the Positive Regulatory Role of Ferritin in Testis Development in the Oriental River Prawn, *Macrobrachium Nipponense*. Front. Physiol..

[3] Sun M., Li X.F., Ge Y.P., Zhang L., Liu B., Liu W.B. (2022). Dietary thiamine requirement and its effects on glycolipid metabolism in oriental river prawn (*Macrobrachium nipponense*). Aquaculture.

[4] Wang L., Feng J.B., Wang G.L., Guan T.Y., Zhu C.K., Li J.L., Wang H. (2021). Effects of cadmium on antioxidant and non-specific immunity of *Macrobrachium nipponense*. Ecotox. Environ. Saf..

[5] Jiang S.F., Zhang W.Y., Xiong Y.W., Cheng D., Wang J.S., Jin S.B., Gong Y.S., Wu Y., Qiao H., Fu H.T. (2022). Hepatopancreas transcriptome analyses provide new insights into the molecular regulatory mechanism of fast ovary maturation in *Macrobrachium nipponense*. BMC Genom..

[6] Qiao H., Jiang F.W., Xiong Y.W., Jiang S.F., Fu H.T., Li F., Zhang W.Y., Sun S.M., Jin S.B., Gong Y.S. (2018). Characterization, expression patterns of molt-inhibiting hormone gene of *Macrobrachium nipponense* and its roles in molting and growth. PLoS ONE.

[7] Qiao H., Xiong Y.W., Jiang S.F., Zhang W.Y., Xu L., Jin S.B., Gong Y.S., Wu Y., Fu H.T. (2020). Three neuroparsin genes from oriental river prawn, *Macrobrachium nipponense*, involved in ovary maturation. 3 Biotech.

[8] Li K., Jia Q.Q., Li S. (2019). Juvenile hormone signaling - a mini review. Insect Sci..

[9] Li Z.Q., Song J.B., Jiang G.H., Shang Y.Z., Jiang Y., Zhang J.F., Xiao L., Chen M., Tang D.M., Tong X.L. (2023). Juvenile hormone suppresses the FoxO-takeout axis to shorten longevity in male *silkworm*. Pestic. Biochem. Phys..

[10] Hyde C.J., Elizur A., Ventura T. (2019). The crustacean ecdysone cassette: A gatekeeper for molt and metamorphosis. J. Steroid Biochem..

[11] Da Silva R.C., do Nascimento F.S., Wenseleers T., Oi C.A. (2022). Juvenile hormone modulates hydrocarbon expression and reproduction in the german wasp *Vespula germanica*. Front. Ecol. Evol..

[12] Liu Z.Y., Huang Z.S., Zheng X.Y., Zheng Z.H., Yao D.F., Zhang Y.L., Aweya J.J. (2022). The juvenile hormone epoxide hydrolase homolog in *Penaeus vannamei* plays immune-related functions. Dev. Comp. Immunol..

[13] Maxwell R.A., Welch W.H., Schooley D.A. (2002). Juvenile Hormone Diol Kinase: I. Purification, characterization, and substrate specificity of juvenile hormone-selective diol kinase from manduca sexta. J. Biol. Chem..

[14] Tusun A., Li M., Liang X.Z., Yang T., Yang B., Wang G. (2017). Juvenile Hormone Epoxide Hydrolase: A Promising Target for Hemipteran Pest Management. Sci. Rep..

[15] Touhara K., Prestwich G.D. (1993). Juvenile hormone epoxide hydrolase. Photoaffinity labeling, purification, and characterization from *tobacco hornworm* eggs. J. Biol. Chem..

[16] Keiser K.C., Brandt K.S., Silver G.M., Wisnewski N. (2002). Cloning, partial purification and in vivo developmental profile of expression of the juvenile hormone epoxide hydrolase of *Ctenocephalides felis*. Arch. Insect Biochem..

[17] Zhang Q.R., Xu W.H., Chen F.S., Li S. (2005). Molecular and biochemical characterization of juvenile hormone epoxide hydrolase from the silkworm, *Bombyx mori*. Insect Biochem. Molec..

[18] Tsubota T., Nakakura T., Shiotsuki T. (2010). Molecular characterization and enzymatic analysis of juvenile hormone epoxide hydrolase genes in the red flour beetle *Tribolium castaneum*. Insect Mol. Biol..

[19] Kamita S.G., Yamamoto K., Dadala M.M., Pha K., Morisseau C., Escaich A., Hammock B.D. (2013). Cloning and characterization of a microsomal epoxide hydrolase from *Heliothis virescens*. Insect Biochem. Molec..

[20] Zhao J., Zhou Y.L., Li X., Cai W.L., Hua H.X. (2017). Silencing of juvenile hormone epoxide hydrolase gene (Nljheh) enhances short wing formation in a macropterous strain of the brown planthopper, *Nilaparvata lugens*. J. Insect Physiol..

[21] Qiao H., Fu H.T., Xiong Y.W., Jiang S.F., Zhang W.Y., Sun S.M., Jin S.B., Gong Y.S., Wang Y.B., Shan D.Y. (2017). Molecular insights into reproduction regulation of female Oriental River prawns *Macrobrachium nipponense* through comparative transcriptomic analysis. Sci. Rep..

[22] Guo B., Wang F., Li Y., Dong S.L. (2013). Effect of periodic light intensity change on the molting frequency and growth of *Litopenaeus vannamei*. Aquac..

[23] Srisala J., Thaiue D., Saguanrut P., Taengchaiyaphum S., Flegel T.W., Sritunyalucksana K. (2023). Wenzhou prawn virus 8 (WzSV8) detection by unique inclusions in prawn hepatopancreatic E-cells and by RT-PCR. Aquaculture.

[24] Livak K.J., Schmittgen T.D. (2001). Analysis of relative gene expression data using real-time quantitative PCR and the 2^− ΔΔCT^ method. Methods.

[25] Chaitanya B.N., Asokan R., Sita T., Rebijith K.B., Ram Kumar P., Krishna Kumar N.K. (2017). Silencing of JHEH and EcR genes of *Plutella xylostella (Lepidoptera: Plutellidae)* through double stranded RNA oral delivery. J. Asia-Pac. Entomol..

[26] Ferreira H.M., Di Pietro V.D., Wenseleers T., Oi C.A. (2023). Conserved role of juvenile hormone in regulating behavioural maturation and division of labour in a highly eusocial wasp. Anim. Behav..

[27] Zhou K., Jia N., Hu C., Jiang Y.L., Yang J.P., Chen Y.X., Li S., Li W.F., Zhou C.Z. (2014). Crystal structure of juvenile hormone epoxide hydrolase from the silkworm *Bombyx mori*. Protein. Sci..

[28] Jing Y.P., Wen X.P., Li L.J., Zhang S.J., Zhang C., Zhou S.T. (2021). The vitellogenin receptor functionality of the migratory locust depends on its phosphorylation by juvenile hormone. Proc. Natl. Acad. Sci. USA..

[29] Borovsky D., Breyssens H., Buytaert E., Peeters T., Laroye C., Stoffels K., Rouge P. (2022). Cloning and Characterization of *Drosophila melanogaster Juvenile Hormone Epoxide Hydrolases (JHEH)* and Their Promoters. Biomolecules.

[30] Borovsky D., Van Ekert E., Buytaert E., Peeters T., Rouge P. (2023). Cloning and characterization of *Aedes aegypti* juvenile hormone epoxide hydrolases (JHEHs). Arch. Insect Biochem..

[31] Wen R.R., Wang B.Y., Wang B.W., Ma L. (2018). Characterization and Expression Profiles of Juvenile Hormone Epoxide Hydrolase from *Lymantria dispar* (Lepidoptera: Lymantridae) and RNA Interference by Ingestion. J. Insect Sci..

[32] Yu Y.H., Hu L.H., Tian D.D., Yu Y.Y., Lu L.Z., Zhang J.M., Huang X.K., Yan M.C., Chen L.B., Wu Z.C. (2023). Toxicities of polystyrene microplastics (MPs) and hexabromocyclododecane (HBCD), alone or in combination, to the hepatopancreas of the whiteleg prawn, *Litopenaeus vannamei*. Environ. Pollut..

[33] Xu Z.N., Liu A., Li S.K., Wang G.Z., Ye H.H. (2020). Hepatopancreas immune response during molt cycle in the mud crab, *Scylla paramamosain*. Sci. Rep..

[34] Liu M.M., Pan J., Liu Z.J., Cheng Y.X., Gong J., Wu X.G. (2018). Effect of estradiol on vitellogenesis and oocyte development of female swimming crab, *Portunus trituberculatus*. Aquaculture.

[35] Chen X.F., Gao Q., Cheng H.H., Peng F., Wang C.L., Xu B.P. (2021). Molecular cloning and expression pattern of the juvenile hormone epoxide hydrolase gene from the giant freshwater prawn *Macrobrachium rosenbergii* during larval development and the moult cycle. Aquac. Res..

[36] Parthasarathy R., Sun Z.Y., Bai H., Palli S.R. (2010). Juvenile hormone regulation of vitellogenin synthesis in the red flour beetle, *Tribolium castaneum*. Insect Biochem. Molec..

[37] Taub-MontemayOr T.E., Rankin M.A.N.N. (1997). Regulation of vitellogenin synthesis and uptake in the boll weevil, *Anthonomus grandis*. Physiol. Entomol..

[38] Caroci A.S., Li Y., Noriega F.G. (2004). Reduced juvenile hormone synthesis in mosquitoes with low teneral reserves reduces ovarian previtellogenic development in *Aedes aegypti*. J. Exp. Biol..

[39] Sheng Z.T., Xu J.J., Bai H., Zhu F., Palli S.R. (2011). Juvenile hormone regulates vitellogenin gene expression through insulin-like peptide signaling pathway in the red flour beetle, *Tribolium castaneum*. J. Biol. Chem..

[40] Yang M.J., Li G.Q., Yu L., Du S.J., Jiang D., Chu X., Wang K., Wu S.Q., Wang R., Zhang F. (2023). Temperature and metal ions regulate larval diapause termination via the 20-hydroxyecdysone and juvenile hormone pathways in *Monochamus alternatus*. Pest Manag. Sci..

[41] Lu F.G., Fu K.Y., Guo W.C., Li G.Q. (2015). Characterization of two juvenile hormone epoxide hydrolases by RNA interference in the *Colorado potato beetle*. Gene.

[42] Zhang X.X., Yuan J.B., Zhang X.J., Xiang J.H., Li F.H. (2020). Genomic Characterization and Expression of Juvenile Hormone Esterase-Like Carboxylesterase Genes in Pacific White Prawn, *Litopenaeus vannamei*. Int. J. Mol. Sci..

